# Astrovascular coupling in the somatosensory cortex of an animal model of amyloidosis at rest and during ambulation

**DOI:** 10.1002/alz.095118

**Published:** 2025-01-09

**Authors:** Ruei‐Lung Lin, Sophiya L Sims, Nicholas A Wright, Leopoldine B Galopin, Olivier Thibault

**Affiliations:** ^1^ University of Kentucky College of Medicine, Lexington, KY USA

## Abstract

**Background:**

We have been investigating *in vivo* astrocytic Ca^2+^ homeostasis in the primary somatosensory cortex (S1) of awake, head‐restrained ambulating mice using two‐photon technology. Prior results from our lab were obtained in neurons across aging, and in male and female C57Bl6/J mice (Case et al., 2023). Here we shifted our attention to studies of astrovascular coupling in the 5xFAD animal model of amyloidosis. S1 neuronal and astrocytic activity is recruited during movement likely through feedback sensory engagement and, as we have previously shown, is sensitive to both sex and age where older females that perform better on ambulatory tasks, also display signs of enhanced neuronal activity with reduced neuronal synchronicity of the network.

**Method:**

Here, we present data from 4 male young‐adult (3 months old) per genotype (IR^fl/fl^ / 5xFAD^+/−^; IR^fl/fl^ / 5xFAD^−/−^). All mice used were derived in house to have double‐floxed insulin receptor alleles and were either WT or 5xFAD (no AAV‐cre injection). All animals received GCaMP8f (AAV5‐Gfa104‐GCaMP8f) unilaterally and following a craniotomy, an imaging window and head plates were mounted over the S1. Four weeks post‐surgery, imaging session were conducted on a two‐photon microscope following retro‐orbital rhodamine dextran delivery to image blood vessels. Both channels were acquired simultaneously for each animal during periods of rest or free ambulation (Neurotar Mobile Home Cage). Several structural and correlational approaches were used to investigate changes in functional astrovascular coupling.

**Result:**

While initial measures of vasoreactivity and astrocyte activity did not show significant differences at this age in either genotype tested, young males did show significant reductions in the density of active astrocytes, while a trend was noted for the density of active vascular elements.

**Conclusion:**

In this initial set of experiments, prior to overt Aβ deposition, we find subtle yet significant structural differences with no significant alteration across extracted astrocyte or vascular network analyses. These were based on correlation coefficients between astrocytes and vascular elements with or without inclusion of endfeet, use of continuous wavelet transforms to address astrocyte encoding frequencies, and tested for network differences in synchronization, vasoreactivity, and network size.

